# Ankylosing spondylitis disease activity score is related to NSAID use, especially in patients treated with TNF-α inhibitors

**DOI:** 10.1371/journal.pone.0196281

**Published:** 2018-04-24

**Authors:** Marlies J. G. Carbo, Anneke Spoorenberg, Fiona Maas, Elisabeth Brouwer, Reinhard Bos, Hendrika Bootsma, Eveline van der Veer, Freke Wink, Suzanne Arends

**Affiliations:** 1 Rheumatology and Clinical Immunology, University Medical Center Groningen, University of Groningen, Groningen, the Netherlands; 2 Rheumatology, Medical Center Leeuwarden, Leeuwarden, the Netherlands; 3 Laboratory Medicine, University Medical Center Groningen, University of Groningen, Groningen, the Netherlands; Nihon University School of Medicine, JAPAN

## Abstract

**Background:**

Non-steroidal anti-inflammatory drugs (NSAIDs) are regarded as the cornerstone of conventional treatment for AS. However little is known about concomitant NSAID use during treatment (with TNF-α inhibitors) in daily clinical practice.

**Methods and findings:**

Consecutive patients from the GLAS cohort were included. NSAID use and ASAS-NSAID index were evaluated at group level and at individual patient level during 52 weeks of follow-up. Analyses were stratified for treatment regimen. Generalized estimating equations (GEE) was used to evaluate NSAID use in relation to assessments of disease activity over time. In patients starting TNF-α inhibitors (n = 254), 79% used NSAIDs at baseline and this proportion decreased significantly to 38% at 52 weeks. ASAS-NSAID index also decreased significantly from median 65 to 0. In patients on conventional treatment (n = 139), 74% used NSAIDs at baseline with median ASAS-NSAID index of 50 and this remained stable during follow-up. At each follow-up visit, approximately half of the patients changed their type or dose of NSAIDs. GEE analysis over time showed that NSAID use was associated with AS disease activity score (p<0.05). This relation was more pronounced in patients treated with TNF-α inhibitors compared to conventional treatment (B = 0.825 vs. B = 0.250).

**Conclusions:**

In this observational cohort of established AS patients, there was no difference in baseline NSAID use between patients with and without indication for TNF-α inhibitors. NSAID use decreased significantly after starting TNF-α inhibitors. During conventional treatment, NSAID use remained stable at group level. However, NSAID use changed frequently at individual patient level and was significantly associated with disease activity.

## Introduction

Ankylosing spondylitis (AS) is a chronic inflammatory rheumatic disease that primarily affects the axial skeleton. Non-steroidal anti-inflammatory drugs (NSAIDs) are regarded as the cornerstone of conventional treatment for AS.[1] NSAIDs have shown good reduction of symptoms in 60–80% of the patients.[2] A recent network meta-analysis of randomized controlled trials (RCTs), reporting on the efficacy of different NSAIDs in AS, showed that the majority of available NSAIDs reduce total pain score significantly compared to placebo up to 12 weeks.[3] Besides the positive effect on pain, NSAIDs also reduce the level of acute-phase reactants in the blood of AS patients.[4] Furthermore, a decrease in signal intensity of bone marrow edema of the sacroiliac (SI) joints on MRI was seen after 6 weeks of full dose NSAIDs in newly diagnosed patients with axial spondyloarthritis (SpA).[5] During treatment, disadvantages of NSAIDs such as possible cardiovascular and gastrointestinal side effects should be taken into account, especially in patients with comorbidity and comedication (e.g. anticoagulants).[6,7]

In AS, there is only limited data available comparing the efficacy of continued use of NSAIDs with on demand use. A single RCT studied symptom control and safety of continued versus on demand use of celecoxib and ketoprofen during 2 years of follow-up as a secondary outcome. No differences were found between the groups.[7] A recent Cochrane review on both traditional and COX-2 selective NSAIDs in AS found no difference in harms between NSAIDs and placebo during 12 weeks of follow-up.[8] Conflicting results were published about the effect of continued versus on demand use of NSAIDs on spinal radiographic progression in AS.[9,10]

For over a decade, tumor necrosis factor-alpha (TNF-α) inhibitors are available for AS patients with active disease who have insufficient response to conventional treatment including NSAIDs. TNF-α inhibitors have shown to reduce the clinical signs and symptoms as well as serum levels of CRP and axial inflammation detected on MRI in AS patients with active disease.[11] A head to head comparison between NSAIDs and TNF-α inhibitors on efficacy in treatment naïve patients has never been performed. Additionally, little is known about the additional efficacy of concomitant NSAID use to the treatment of biologicals in AS. Therefore, it can be questioned whether AS patients should be advised to stop or continue their NSAIDs during TNF-α inhibitor use.[5]

So far, studies on concomitant NSAID use to the treatment of biologicals were only performed in patients with early active axiale SpA.[12–15] A recent RCT showed that patients reached partial remission more frequently during treatment with infliximab combined with naproxen than during naproxen treatment alone.[16] Another recent RCT showed the NSAID sparing effect of etanercept treatment. Patients were able to reduce their NSAID intake by more than half during 8 weeks of etanercept treatment.[15] In the observational DESIR cohort, patients were included presenting with inflammatory back pain, symptom duration between ≥3 months and 3 years and symptoms suggestive of spondyloarthritis according to the local investigator. However, these patients did not necessarily fulfill the modified New York criteria for AS.[13] Patients receiving TNF-α inhibitors from this cohort were matched to patients on conventional treatment using propensity scores. After 2 years of follow-up, in both treatment groups the proportion of patients using NSAIDs decreased as well as the Assessment of Spondyloarthritis international Society (ASAS)-NSAID index, which is based on the specific NSAID, dosage and frequency. These results were significantly more pronounced in the group receiving TNF-α inhibitors.[15] Based on these results, it was concluded that most patients can reduce their intake of NSAIDs after starting TNF-α inhibitors. However, not all patients receiving TNF-α inhibitors were able to stop or decrease their NSAID intake.

The aim of the present study was to evaluate NSAID use over time and to investigate if NSAID use was related to disease activity in patients with established AS from a large observational cohort study starting TNF-α inhibitors or receiving conventional treatment during a follow-up period of 52 weeks.

## Methods

### Patients

**Fig 1:**
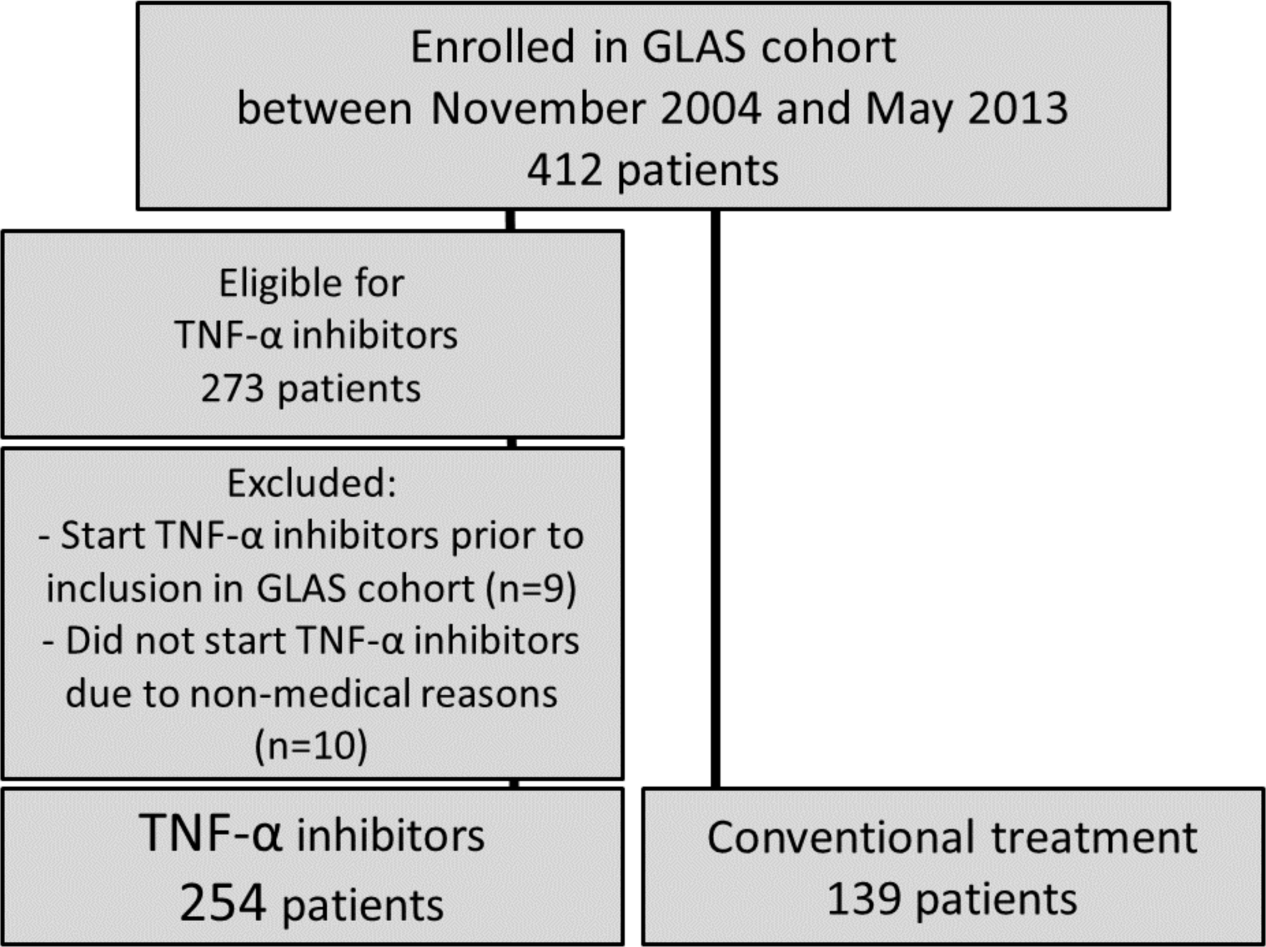
Flowchart of inclusion.

The GLAS cohort was approved by the local ethics committees of the MCL and UMCG and all patients provided written informed consent according to the Declaration of Helsinki. The present analysis was performed in the Groningen Leeuwarden Ankylosing Spondylitis (GLAS) cohort. The GLAS cohort is an ongoing prospective longitudinal observational cohort study with standardized follow-up. Since November 2004, consecutive AS outpatients who started TNF-α inhibitors from the University Medical Center Groningen (UMCG) and the Medical Center Leeuwarden (MCL) were included.[17] In 2009, this inclusion was extended to all consecutive axial SpA outpatients, irrespective of treatment regimen. All patients were 18 years or older and fulfilled the modified New York criteria for AS (>90%) or the ASAS criteria for axial SpA including MRI. All patients included before June 2013 were included in this analysis. Patients were excluded if they started TNF-α inhibitors prior to inclusion in the GLAS cohort or had an indication but did not start with TNF-α inhibitors due to non-medical reasons, such as the patient’s own preference (Fig 1).

### Treatment

NSAIDs were prescribed based on expert opinion. TNF-α inhibitors were started according to the ASAS consensus statement.[18] The choice between available TNF-α inhibitors was based on the judgment of the treating rheumatologist and/or the specific preference of the patient.[17] After 3 months of TNF-α inhibitor treatment, patients with low disease activity were advised to take NSAIDs on demand. There was no difference in treatment advice between males and females.

### Follow-up and clinical assessments

Visits were scheduled at baseline (before start of TNF-α inhibitors) and after 6, 12, 24 and 52 weeks for the TNF-α inhibitor group. The conventional treatment group had scheduled visits at baseline, 24 and 52 weeks. At every visit, disease activity was assessed using the AS Disease Activity Score (ASDAS), Bath AS Disease Activity Index (BASDAI), and serum CRP level. [19–20]

At every visit, NSAID use (yes/no) and type of NSAID was recorded. Dosage and frequency were assessed retrospectively from clinical records to calculate the ASAS-NSAID index. This index takes the specific NSAID and dosage into account as well as the percentage of days of NSAID intake during a period of interest.[1,21] A score of 100 is equal to a full dose of NSAIDs all days of the week. For situations in which the exact amount of NSAID use was unclear, a frequency of 2 days a week was used for occasional NSAID use (1 to 3 days a week) and 0.5 days a week for very rare NSAID use (<1 day a week). To determine the changes in NSAID use, the type and dosage of NSAIDs was assessed at each visit to investigate starting, switching, dose escalation, dose reduction or complete stop of NSAIDs.

### Statistics

Results were expressed as number of patients (%) and mean ± SD or median (interquartile range; IQR), for categorical and normally or non-normally distributed data, respectively. Analyses were stratified for treatment regimen: patients starting TNF-α inhibitors and patients on conventional treatment. Chi-square or Fisher’s Exact tests, Independent Samples t-tests, and Mann-Whitney U-tests were used to compare baseline characteristics. Linear and logistic generalized estimating equations (GEE) were used to analyze NSAID use over time. GEE is a technique for longitudinal data analysis, which makes use of all available data and allows unequal numbers of repeated measurements.[22] The ‘exchangeable’ correlation structure was used to correct for within patient correlation. If there was no normal distribution of residuals, parameters were log-transformed before being entered into the equation.

GEE was also applied to analyze the relation between NSAID use and disease activity over time, where disease activity was defined as the dependent variable. NSAID use was analyzed using 4 parameters: NSAID use (yes versus no), ASAS-NSAID index (continuous scale), low (index ≥10 versus <10), and high use (index ≥90 versus <90). To exclude potential bias due to TNF-α inhibitors discontinuation, analyses were also performed in patients using TNF-α inhibitors ≥80% of the follow up time. To exclude the influence of the initial positive effect of TNF-α inhibitors on the association between NSAID use and disease activity, analyses were also performed for 12 to 52 weeks of follow-up. P-values <0.05 were considered statistically significant. Statistical analysis was performed with IBM SPSS Statistics 22 (SPSS, Chicago, IL, USA).

## Results

Of the 393 included AS patients, 67% were male, mean age was 44 ± 13 years, median symptom duration 15 years (IQR 8–24), and 79% were HLA-B27 positive. Additionally, 254 (66%) patients started TNF-α inhibitors due to active disease (etanercept (58%), adalimumab (26%), or infliximab (15%)). Of the 254 patients who started TNF-α inhibitors, 217 (85%) used this treatment more than 80% of the follow-up time. The remaining 139 (34%) patients received conventional treatment. Patient characteristics were comparable between both groups, except higher disease activity, more often peripheral arthritis, and worse physical functioning in patients starting TNF-α inhibitors.(Table 1)

**Table 1:** Baseline characteristics of the AS study population.

	Total	TNF-α inhibitors	Conventional treatment	P-value*
	n = 393	n = 254	n = 139	
**Patient characteristics**				
Age (yrs)	44 ± 13	43 ± 12	44 ± 14	0.451
Male gender	261 (66%)	173 (68%)	88 (64%)	0.253
HLA-B27+	294 (79%)	197 (81%)	97 (75%)	0.094
Duration of symptoms (yrs)	15 (8–24)	15 (9–24)	14 (7–24)	0.393
Time since diagnosis (yrs)	6 (1–16)	7 (1–24)	5 (1–16)	0.693
**History of extra-spinal manifestations**				
Peripheral arthritis	102 (27%)	76 (31%)	26 (20%)	**0.015**
IBD	36 (10%)	23 (9%)	13 (10%)	0.495
Uveitis	108 (28%)	73 (29%)	35 (27%)	0.334
Psoriasis	28 (7%)	15 (6%)	13 (10%)	0.125
**Disease status**				
BASDAI (range 0 to 10)	5.3 ± 2.2	6.1 ± 1.7	3.9 ± 2.2	**<0.001**
ASDAS	3.3 ± 1.1	3.8 ± 0.8	2.4 ± 0.9	**<0.001**
CRP (mg/l)	8 (8–17)	13 (5–22)	3 (2–7)	**<0.001**
ESR (mm/h)	14 (7–28)	21 (10–35)	9 (4–13)	**<0.001**
Peripheral arthritis	50 (13%)	43 (17%)	7 (5%)	**<0.001**
BASFI (range 0 to 10)	4.8 ± 2.5	5.7 ± 2.1	3.2 ± 2.3	**<0.001**
**Concomitant medication use**				
Current NSAID use	289 (77%)	194 (79%)	95 (74%)	0.131
Current DMARD use	57 (15%)	47 (19%)	10 (7%)	**0.002**

*p-value: TNF-α inhibitors vs. conventional treatment. Values are number of patients (percentage), mean ± SD or median (IQR). HLA-B27+, human leukocyte antigen B27 positive; IBD, inflammatory bowel disease; BASDAI, Bath Ankylosing Spondylitis Disease Activity Index; ASDAS, Ankylosing Spondylitis Disease Activity Score; CRP, C-reactive protein; ESR, erythrocyte sedimentation rate; BASFI, Bath Ankylosing Spondylitis Functional Index; DMARD, disease-modifying antirheumatic drug

### NSAID use at group level

At baseline, 77% of patients used 12 different NSAIDs, mostly diclofenac (16%), naproxen (16%) and piroxicam (7%).(S1 Table) Baseline NSAID use was comparable between patients who started TNF-α inhibitors and patients on conventional treatment (79% vs. 74%). In patients who started TNF-α inhibitors, this proportion decreased significantly during follow-up to 38% at 52 weeks. When analyzing 2 different subgroups, low on demand and high NSAID use (defined by ASAS-NSAID as index resp. <10 and ≥90), the largest decrease in NSAID use was found during the first 6 weeks, followed by a slower gradual decrease up to 52 weeks.(Table 2) In the conventional treatment group, the proportion of patients using NSAIDs remained stable over time (82% at 52 weeks).(Table 2)

**Table 2:** Changes in NSAID use at group level.

	Baseline	6 weeks	12 weeks	24 weeks	52 weeks	p- value
**TNF-α inhibitors**	n = 245	n = 214	n = 223	n = 223	n = 217	
NSAID use (Y/N)	79%	57%	47%	41%	38%	**<0.001**
ASAS-NSAID index	65 (23–100)	25 (0–60)	0 (0–50)	0 (0–50)	0 (0–50)	**<0.001**
NSAID use low	24%	47%	56%	76%	77%	**<0.001**
NSAID use high	44%	21%	15%	16%	14%	**<0.001**
**Conventional treatment**	n = 129			n = 109	n = 112	
NSAID use (Y/N)	74%			80%	82%	0.183
ASAS-NSAID index	50 (0–100)			50 (5–100)	50 (7–100)	0.382
NSAID use low	44%			31%	28%	0.448
NSAID use high	36%			37%	38%	0.721

Values are percentage of patients or median (IQR).

Patients starting TNF-α inhibitors also showed a rapid and significant decrease in ASAS-NSAID index during the first 6 to 12 weeks and this reduction remained stable during all follow-up visits. In patients on conventional treatment, the total ASAS-NSAID index and subgroups did not change over time.(Table 2) These patterns were found in both males and females (data not shown). GEE analysis showed no significant interaction between gender and ASAS-NSAID index over time.

In total, 22 patients (6%) never used NSAIDs, of which 18 patients in the TNF-α inhibitor group and 4 patients in the conventional treatment group. In the TNF-α inhibitor group, 9 patients had known contraindications for NSAID use (gastrointestinal (n = 4), renal (n = 4) and a proven allergy (n = 1)). In the conventional treatment group, there were no known contraindications.

### NSAID use at individual patient level

Assessing the variability in NSAID use at individual patient level revealed that 46 to 67% of the patients treated with TNF-α inhibitors remained on a stable dose at each follow-up visit. Furthermore, 20 to 46% of the patients reduced their dose or stopped NSAIDs at a follow-up visit. Additionally, only 8 to 15% of the patients increased their dose, started or switched NSAIDs at a follow-up visit.(Table 3) Of the patients using NSAIDs at baseline, 48% had stopped their NSAIDs 52 weeks after starting TNF-α inhibitors.

**Table 3:** Changes in NSAID use at individual patient level.

	Baseline	6 weeks	12 weeks	24 weeks	52 weeks
**TNF-α Inhibitors**					
NSAID use at baseline	79%				
Dose escalation, start or switch		8%	11%	13%	15%
Stable dose		46%	59%	67%	61%
Dose reduction or stop		46%	30%	20%	24%
**Conventional treatment**					
NSAID use at baseline	74%				
Dose escalation, start or switch				34%	26%
Stable dose				46%	49%
Dose reduction or stop				21%	26%

Patients who used no NSAIDs during follow-up were excluded from this table: 11% in TNF-α Inhibitor group and 6% in the conventional treatment group.

Although NSAID use at group level was stable over time in the conventional treatment group, there were many changes at individual patient level. At each follow-up visit, approximately half of the patients changed their NSAID use: 26 to 34% of the patients showed dose escalation, start or switch of NSAIDs and 21 to 26% dose reduction or stop of NSAIDs.(Table 3) Only 7% used NSAIDs at baseline and not at 52 weeks, where 13% did not use NSAIDs at baseline but had started at 52 weeks of follow up.

### NSAID use in relation to disease activity

**Fig 2:**
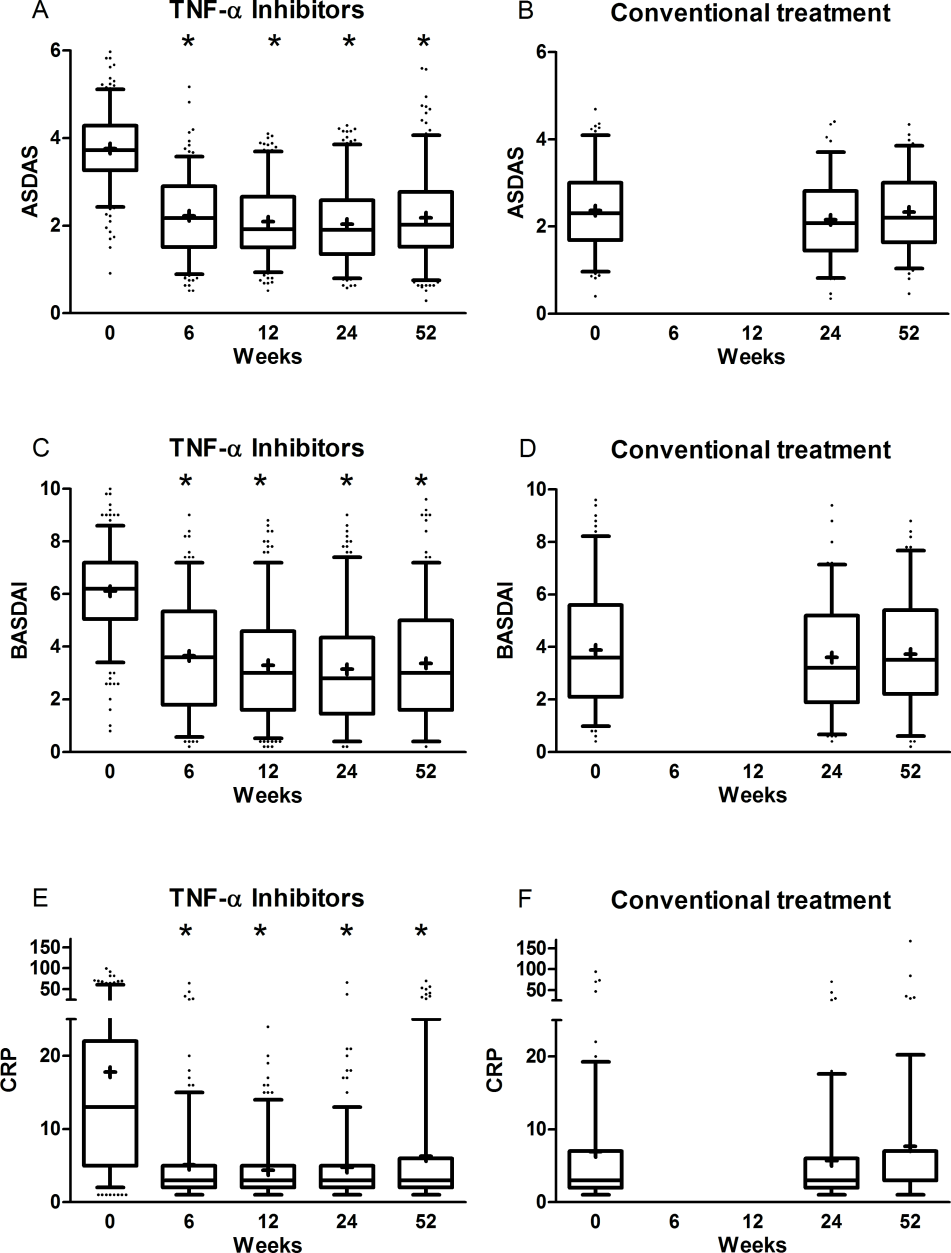
Disease activity over time in AS patients. ASDAS over time in patients starting TNF-α inhibitors (A) and receiving conventional treatment (B); BASDAI over time in patients starting TNF-α inhibitors (C) and receiving conventional treatment (D); CRP over time in patients starting TNF-α inhibitors (E) and receiving conventional treatment (F). *p<0.05 compared to baseline. Box-and-whisker plots: boxes indicate medians with interquartile ranges; + indicate means; whiskers indicate 5–95 percentile; • indicate outliers.

Disease activity reduced significantly after starting TNF-α inhibitors and remained low and stable during follow-up. In the conventional treatment group, disease activity was low and stable at all visits.(Fig 2) At 52 weeks, disease activity was comparable between both groups, BASDAI mean 3.4 vs. 3.7, ASDAS mean 2.2 vs. 2.3, and CRP median 3 vs. 3 respectively.

GEE analysis over time showed that NSAID use was significantly associated with disease activity. In the TNF-α inhibitor group, a significant association of all 4 NSAID parameters (NSAID use (yes/no), ASAS-NSAID index, low on demand use and high use) with ASDAS was found. GEE revealed that higher NSAID use was related to higher ASDAS and vice versa.(Table 4) Comparable results were found for BASDAI and CRP.(S2 and S3 Tables)

**Table 4:** Association between ASDAS and NSAID use over time in AS patients.

	B (95% CI)	P-value	Interval	n
**TNF-α Inhibitors**				
NSAID use Yes	0.825 (0.664–0.985)	**<0.001**	1074	251
ASAS-NSAID index	0.009 (0.007–0.012)	**<0.001**	1073	251
NSAID use low	-0.831 (-0.672- -0.990)	**<0.001**	1073	251
NSAID use high	0.855 (0.682–1.028)	**<0.001**	1073	251
**Conventional treatment**				
NSAID use Yes	0.250 (0.006–0.493)	**0.045**	315	131
ASAS-NSAID index	0.002 (0.000–0.005)	0.059	314	131
NSAID use low	- 0.223 (-0.425–0.022)	**0.030**	314	131
NSAID use high	0.269 (0.038–0.501)	**0.023**	314	131

The association between NSAID use and ASDAS remained significant in patients who used TNF-α inhibitors more than 80% of the follow-up time and when analyzing only 12 to 52 weeks of follow-up to exclude the initial effect of TNF-α inhibitors, although the regression coefficients were lower in these last analyses. (S4 Table)

In the conventional treatment group, a significant but less prominent association of 3 NSAID parameters NSAID use (yes/no), low on demand use and high use) with ASDAS was found. (Table 4) BASDAI was only significantly associated with low on demand NSAID use. For CRP, no significant associations with NSAID use were found.

## Discussion

In the present prospective observational cohort study, we evaluated NSAID use in relation to disease activity during 52 weeks of follow-up in established AS patients treated with and without TNF-α inhibitors in daily clinical practice. This is the first study assessing the difference in NSAID use at group level and at individual patient level in established AS.

In the TNF-α inhibitor group, a rapid and significant decrease of concomitant NSAID use was seen. NSAID use at group level remained low during the entire follow-up period. Although patients in our cohort were advised to take their NSAID on demand after 3 months of TNF-α inhibitor treatment, many patients reduced or stopped NSAIDs on their own initiative even earlier. The reason for this is most likely the positive effect on symptoms of TNF-α inhibitors. Our finding of rapid NSAID reduction after 6–12 weeks of TNF-α inhibitor treatment is in line with a previous RCT in early axial SpA which showed the NSAID sparing effect of 8 weeks etanercept treatment.[15] Additionally, early axial SpA patients of the DESIR cohort showed a gradual decline in NSAID use after starting TNF-α inhibitors during 2 years of follow-up.[14]

In our conventional treatment group, NSAID use remained stable over 52 weeks of follow-up, whereas in the conventional care group of the DESIR cohort a significant decrease in NSAID use over 2 years was shown.[14] The GLAS cohort is an established AS cohort in contrast to the DESIR cohort, which includes patients with recent-onset (<3 years) inflammatory back pain suggestive of axial SpA.[14] Furthermore, DESIR used propensity scores to match the conventional treatment group to the TNF-α inhibitor group. This may explain the differences in the course of NSAID use between the conventional treatment groups of both cohorts. In concordance with our results, in the DESIR cohort the decrease in NSAID use of patients on conventional treatment was also less pronounced than in patients starting TNF-α inhibitor group.[14]

In our GLAS cohort, frequent inter-individual changes in NSAID use were observed at each follow-up visit in both treatment groups. Interestingly, GEE analysis over time showed that NSAID use was significantly associated with disease activity. In the TNF-α inhibitor group, this relation was most pronounced and significant for all disease activity measures (i.e. ASDAS, BASDAI and CRP). Results remained significant when including patients who used TNF-α inhibitors more than 80% of the follow-up time and when analyzing 12 to 52 weeks of follow-up to exclude the influence of the initial positive effect of TNF-α inhibitors on the GEE analyses.

In the conventional treatment group, the association between NSAID use and disease activity was less pronounced and only found for ASDAS. This can be explained by less variation in disease activity in this treatment group, e.g. the large majority of patients had normal CRP levels, but it can also be related to lower power in GEE analysis because of the lower number of included patients and less frequent follow-up visits.

Since this is an observational cohort study in AS patients, it reflects NSAID use in daily clinical practice. No sub-analyses were performed on specific NSAIDs, e.g. traditional and COX-2 NSAIDs, due to small numbers. However, a recent meta-analysis showed no clear superiority in efficacy of any specific NSAID.[3] Unfortunately, systematic data on the side effects of NSAIDs were missing in our study, which potentially could have explained why patients reduced or stopped their NSAID intake. Despite this limitation, a positive association between NSAID use and disease activity was still found.

In summary, this is the first study investigating NSAID use in an established AS cohort on TNF-α inhibitors or conventional treatment. At baseline, we found no differences in NSAID use between AS patients with and without indication for TNF-α inhibitors. After starting TNF-α inhibitors, NSAID use decreased rapidly and remained low during follow-up. In patients receiving conventional treatment, NSAID use remained stable at group level over time. Conversely, at individual patient level, both the type and dose of NSAIDs changed frequently irrespective of treatment regimen. Therefore, stable NSAID use at group level does not necessarily reflect stable NSAID use at individual patient level. This should be taken into account in future evaluations and research on NSAID use in axial SpA. Most importantly, this is the first prospective cohort study of established AS patients that showed NSAID use was significantly associated with assessments of disease activity, which was most pronounced for patients treated with TNF-α inhibitors.

## Supporting information

S1 Table Specific NSAIDs used at baseline. (DOCX)

S2 Table Association between BASDAI and NSAID use over time in AS patients. *Subgroup analysis of patients who used TNF-α inhibitors ≥80% of the follow up time. ** Analysis for 12 to 52 weeks of follow-up (excluding baseline and 6 weeks).
(DOCX)

S3 Table Association between CRP and NSAID use over time in AS patients. *Subgroup analysis of patients who used TNF-α inhibitors ≥80% of the follow up time. **Analysis for 12 to 52 weeks of follow-up (excluding baseline and 6 weeks).
(DOCX)

S4 Table Association between ASDAS and NSAID use over time in AS patients. *Subgroup analysis of patients who used TNF-α inhibitors ≥80% of the follow up time. ** Analysis for 12 to 52 weeks of follow-up (excluding baseline and 6 weeks).
(DOCX)
