# Supplementary material for: Ankylosing spondylitis disease activity score is related to NSAID use, especially in patients treated with TNF-α inhibitors
Source: PLoS One. 2018 Apr 24;13(4):e0196281. doi: 10.1371/journal.pone.0196281 (PMC5915774; doi:10.1371/journal.pone.0196281)
Supplement: S1 Table — (DOCX) [file pone.0196281.s001.docx]

**S1 Table. Specific NSAIDs used at baseline.**

| **NSAID** | **Frequency** | **Percentage** |
| --- | --- | --- |
| Diclofenac | 61 | 15.5 |
| Naproxen | 61 | 15.5 |
| Piroxicam | 29 | 7.4 |
| Ibuprofen | 22 | 5.6 |
| Celecoxib | 21 | 5.3 |
| Indomethacin | 21 | 5,3 |
| Etoricoxib | 20 | 5.1 |
| Fenylbutazone | 20 | 5.1 |
| Meloxicam | 19 | 4.8 |
| Butazolidine | 5 | 1.3 |
| Ketaprofen | 5 | 1.3 |
| Namubetone | 1 | 0.3 |
| No NSAID | 79 | 20.1 |
| Total | 393 | 100 |
