# Supplementary material for: Ankylosing spondylitis disease activity score is related to NSAID use, especially in patients treated with TNF-α inhibitors
Source: PLoS One. 2018 Apr 24;13(4):e0196281. doi: 10.1371/journal.pone.0196281 (PMC5915774; doi:10.1371/journal.pone.0196281)
Supplement: S2 Table — *Subgroup analysis of patients who used TNF-α inhibitors ≥80% of the follow up time. ** Analysis for 12 to 52 weeks of follow-up (excluding baseline and 6 weeks). (DOCX) [file pone.0196281.s002.docx]

**S2 Table. Association between BASDAI and NSAID use over time in AS patients.**

|  |  | **B (95% CI)** | **P-value** | **Interval** | **n** |
| --- | --- | --- | --- | --- | --- |
| **TNF-α Inhibitors** |  | | | | |
| NSAID use Yes | Complete group | 1.563 (1.250-1.875) | **<0.001** | 1086 | 251 |
|  | TNF-α ≥80%* | 1.790 (1.474-2.106) | **<0.001** | 967 | 214 |
|  | 12-52 weeks** | 0.549 (0.207-0.891) | **0.002** | 632 | 246 |
| ASAS-NSAID index | Complete group | 0.017 (0.012-0.021) | **<0.001** | 1084 | 251 |
|  | TNF-α ≥80%* | 0.019 (0.015-0.024) | **<0.001** | 965 | 214 |
|  | 12-52 weeks** | 0.008 (0.003-0.013) | **0.001** | 631 | 246 |
| NSAID use low | Complete group | -1.577 (-1.884--1.269) | **<0.001** | 1084 | 251 |
|  | TNF-α ≥80%* | -1.756 (-2.067--1.444) | **<0.001** | 965 | 214 |
|  | 12-52 weeks** | -0.564 (-0.920--0.208) | **0.002** | 631 | 246 |
| NSAID use high | Complete group | 1.571 (1.238-1.904) | **<0.001** | 1084 | 251 |
|  | TNF-α ≥80%* | 1.690 (1.341-2.040) | **<0.001** | 965 | 214 |
|  | 12-52 weeks** | 0.855 (0.415-1.294) | **<0.001** | 631 | 246 |
| **Conventional treatment** |  | | | | |
| NSAID use Yes |  | 0.165 (-0.317-0.646) | 0.503 | 333 | 132 |
| ASAS-NSAID index |  | 0.003 (-0.001-0.007) | 0.093 | 332 | 132 |
| NSAID use low |  | -0.326 (0.749--0.097) | 0.131 | 332 | 132 |
| NSAID use high |  | 0.514 (0.073-0.955) | **0.022** | 332 | 132 |

*Subgroup analysis of patients who used TNF-α inhibitors ≥80% of the follow up time. ** Analysis for 12 to 52 weeks of follow-up (excluding baseline and 6 weeks).
