# Supplementary material for: Ankylosing spondylitis disease activity score is related to NSAID use, especially in patients treated with TNF-α inhibitors
Source: PLoS One. 2018 Apr 24;13(4):e0196281. doi: 10.1371/journal.pone.0196281 (PMC5915774; doi:10.1371/journal.pone.0196281)
Supplement: S3 Table — *Subgroup analysis of patients who used TNF-α inhibitors ≥80% of the follow up time. **Analysis for 12 to 52 weeks of follow-up (excluding baseline and 6 weeks). (DOCX) [file pone.0196281.s003.docx]

**S3 Table. Association between CRP and NSAID use over time in AS patients.**

|  |  | **B (95% CI)** | **P-value** | **Interval** | **n** |
| --- | --- | --- | --- | --- | --- |
| **TNF-α Inhibitors** |  |  |  |  |  |
| NSAID use Yes | Complete group | 4.624 (3.131-6.118) | **<0.001** | 1079 | 251 |
|  | TNF-α ≥80%* | 5.175 (3.540-6.810) | **<0.001** | 957 | 214 |
|  | 12-52 weeks** | 1.707 (-0.008-3.807) | 0.051 | 636 | 247 |
| ASAS-NSAID index | Complete group | 0.051 (0.032-0.070) | **<0.001** | 1078 | 251 |
|  | TNF-α ≥80%* | 0.063 (0.042-0.084) | **<0.001** | 956 | 214 |
|  | 12-52 weeks** | 0.034 (0.005-0.062) | **0.020** | 635 | 247 |
| NSAID use low | Complete group | -4.801 (-6.361--3.242) | **<0.001** | 1078 | 251 |
|  | TNF-α ≥80%* | -5.402 (-7.118--3.687) | **<0.001** | 956 | 214 |
|  | 12-52 weeks** | -1.603 (-3.414--0.209) | 0.083 | 635 | 247 |
| NSAID use high | Complete group | 4.573 (2.634-6.513) | **<0.001** | 1078 | 251 |
|  | TNF-α ≥80%* | 5.483 (3.307-7.659) | **<0.001** | 956 | 214 |
|  | 12-52 weeks** | 3.806 (0.918-6.694) | **0.010** | 635 | 247 |
| **Conventional treatment** |  | | | | |
| NSAID use Yes |  | -0.914 (-2.828-1.000) | 0.349 | 328 | 132 |
| ASAS-NSAID index |  | -0.003 (-0.019-0.013) | 0.700 | 328 | 132 |
| NSAID use low |  | 0.582 (0.931-2.095) | 0.451 | 328 | 132 |
| NSAID use high |  | -0.075 (-1.627-1.477) | 0.924 | 328 | 132 |

*Subgroup analysis of patients who used TNF-α inhibitors ≥80% of the follow up time. **Analysis for 12 to 52 weeks of follow-up (excluding baseline and 6 weeks).
