# Supplementary material for: Ankylosing spondylitis disease activity score is related to NSAID use, especially in patients treated with TNF-α inhibitors
Source: PLoS One. 2018 Apr 24;13(4):e0196281. doi: 10.1371/journal.pone.0196281 (PMC5915774; doi:10.1371/journal.pone.0196281)
Supplement: S4 Table — *Subgroup analysis of patients who used TNF-α inhibitors ≥80% of the follow up time. ** Analysis for 12 to 52 weeks of follow-up (excluding baseline and 6 weeks). (DOCX) [file pone.0196281.s004.docx]

**S4 Table. Association between ASDAS and NSAID use over time in AS patients.**

|  |  | **B (95% CI)** | **P-value** | **Interval** | **n** |
| --- | --- | --- | --- | --- | --- |
| **TNF-α Inhibitors** |  | | | | |
| NSAID use Yes | Complete group | 0.825 (0.664-0.985) | **<0.001** | 1074 | 251 |
|  | TNF-α ≥80%* | 0.927 (0.762-1.091) | **<0.001** | 954 | 214 |
|  | 12-52 weeks** | 0.330 (0.169-0.490) | **<0.001** | 634 | 246 |
| ASAS-NSAID index | Complete group | 0.009 (0.007-0.012) | **<0.001** | 1073 | 251 |
|  | TNF-α ≥80%* | 0.011 (0.009-0.013) | **<0.001** | 953 | 214 |
|  | 12-52 weeks** | 0.005 (0.003-0.007) | **<0.001** | 633 | 246 |
| NSAID use low | Complete group | -0.831 (-0.990--0.672) | **<0.001** | 1073 | 251 |
|  | TNF-α ≥80%* | -0.920 (-1.085--0.755) | **<0.001** | 953 | 214 |
|  | 12-52 weeks** | -0.324 (-0.488--0.161) | **<0.001** | 633 | 246 |
| NSAID use high | Complete group | 0.855 (0.682-1.028) | **<0.001** | 1073 | 251 |
|  | TNF-α ≥80%* | 0.935 (0.750-1.121) | **<0.001** | 953 | 214 |
|  | 12-52 weeks** | 0.514 (0.304-0.724) | **<0.001** | 633 | 246 |

*Subgroup analysis of patients who used TNF-α inhibitors ≥80% of the follow up time. ** Analysis for 12 to 52 weeks of follow-up (excluding baseline and 6 weeks).
